# Does work modify the relationship between violence and mental health among young people? Evidence from the Violence Against Children Surveys in Uganda, Nigeria and Colombia

**DOI:** 10.7189/jogh.14.04232

**Published:** 2024-11-22

**Authors:** Jodie Pearlman, Harriet Morgan, Charles Opondo, Mathew Amollo, Jorge Cuartas, Amiya Bhatia

**Affiliations:** 1 Department of Population Health, London School of Hygiene and Tropical Medicine, London, UK; 2 Department of Medical Statistics, London School of Hygiene and Tropical Medicine, London, UK; 3 AfriChild Centre, Makerere University, Kampala, Uganda; 4 Department of Applied Psychology, New York University, New York, USA; 5 Centro de Estudios sobre Seguridad y Drogas (CESED), Universidad de los Andes, Bogotá, Colombia; 6 Department of Social Policy and Intervention, University of Oxford, Oxford, UK

## Abstract

**Background:**

Considering the well-established link between experiencing violence in childhood and poor mental health, it is important to understand the role of contextual factors in which young people live, learn, work, and play. Evidence has highlighted the importance of work as a contextual factor in the lives of young people, but it is unclear whether being in work mitigates, entrenches, or exacerbates the association between violence against children and poor mental health.

**Methods:**

This study is based on nationally representative data of males and females aged 13–24 years who completed the Violence Against Children Surveys in Nigeria, Uganda, and Colombia. We used multivariable logistic regression models to explore the association between lifetime violence and past 30-day mental distress, and explored whether working in the past year modified this association. All analyses were age- and sex-stratified and conducted separately in each country.

**Results:**

We found a high prevalence of lifetime violence among young people in all three countries and strong associations between violence and mental distress. In Colombia, there was strong evidence (*P*-value for interaction = 0.014) that work in the past year (adjusted odds ratio (aOR) = 0.42; 95% confidence interval (CI) = 0.07–2.57) reduced the risk of mental distress among girls who had experienced violence compared to not working in the past year (aOR = 6.12; 95% CI = 2.60–14.41). There was also evidence among boys in Nigeria (*P*-value for interaction = 0.045), where there was a reduction in risk of mental distress among those not in work in the past year (aOR = 0.99; 95% CI = 0.46–2.13) compared to those in work in the past year (aOR = 2.10; 95% CI = 1.32–3.33). There was also a pattern of effect modification by work in the association between childhood violence and mental health in other population groups, with variation by country, sex, and age groups.

**Conclusions:**

Our findings suggest that work can mitigate, entrench, or exacerbate risk of mental distress among young people who have experienced violence, depending on their age, sex, and country of residence. While additional research is needed to explore these mechanisms, this highlights the importance of work as a contextual factor in the lives of young people and points to the need for safe and secure workplaces and the integration of context-specific interventions into workplaces for young people.

Global estimates suggest that one in two children aged 2–17 years experience violence each year, equivalent to around one billion children, with the highest rates observed in Africa, Asia, and North America [1]. Substantial evidence highlights the relationship between experiencing childhood violence and poor mental health [2–7]. A systematic review of 30 studies in low- and lower-middle-income countries found that experiencing violence in childhood increases the likelihood of developing several mental disorders, including posttraumatic stress disorder, depression and anxiety, and developmental problems, with the greatest risk among children who experienced multiple forms of violence or polyvictimisation [8]. Another systematic review of 96 studies found similar evidence that experiencing childhood physical and emotional abuse and neglect increased the risk of a range of mental health disorders, including depression, anxiety, and suicidal behaviour [7].

While the link between experiencing childhood violence and poor mental health is well-established, there is a need to understand how the contexts in which young people live, learn, work, and play may mitigate, entrench, or exacerbate the risk of poor mental health in order to guide the development of effective interventions and policies. The socioecological model highlights the individual, relationship, community, and societal contexts surrounding an individual and can therefore be used to conceptualise the factors in the home, school, neighbourhood, and workplace that may influence the relationship between violence and mental health [9,10]. Although existing literature has explored how family relationships and community- and school-related factors influence the risk of developing poor mental health following experience of childhood violence, there is limited research into the role of work as a contextual factor. A longitudinal UK study among 2–9-year-old children exposed to parental intimate partner violence found that those who had a good relationship with peers, enjoyed school, and lived in safer and cohesive neighbourhoods had a reduced risk of depressive symptoms at 18 years old [11]. Another UK study among adults suggested that having support from a trusted adult during childhood may mitigate the negative mental health impacts associated with adverse childhood experiences [12]. Evidence has also suggested that having a stable family environment and supportive relationships is associated with development of resilience following experience of child maltreatment [13]. Research in the USA and Canada has suggested that having good coping skills, positive self-esteem and an internal locus of control, doing physical activity, and obtaining higher education may all mitigate the relationship between child maltreatment and poor mental health [14,15]. Overall, the evidence draws attention to the role of individual-level factors, and factors related to family relationships and experiences in school and the community, in mitigating poor mental health among those who have experienced violence in childhood. Conversely, although work, alongside schools and neighbourhoods, is an important community-level factor that may influence the risk of poor mental health among those who experience violence in childhood, little research has explored the importance of work in influencing this relationship. A study in Canada found that earning a higher income may reduce the risk of poor mental health among adults who experienced child abuse [14]. However, to our knowledge, no research has explored whether being in work can mitigate or exacerbate the relationship between childhood violence and mental health.

Work plays an important role in the lives of young people and can impact them in both positive and negative ways, depending on their age, the sector and type of work, and the relationship between work and other contextual factors such as education. Evidence suggests that 483 million young people aged 15–24 years are in the labour force, either employed or unemployed. Young people may work in either paid or unpaid positions, and across a range of sectors within either formal or informal employment, including domestic work, agriculture and economic activities, or more hazardous sectors such as mining and construction [16].

Existing research has highlighted links between work and mental health, though these associations vary depending on the nature of work. For example, existing evidence suggests that employment is associated with improved general mental health and depression [17] and that earning a higher income is associated with improved mental health, particularly for those in poverty or in low- and middle-income countries [18]. Individuals in insecure or precarious employment were also found to have a higher risk of poor mental health [19,20]. The majority of research exploring these links has been conducted in high-income settings, where the nature of work is likely to vary to lower-income settings. Work also plays a different role for children compared to young people over 18 years. For young people, the transition into work is an important stage in entering adulthood, as it enables them to earn an income and gain economic independence and make connections within society, and young people who are unemployed have higher risks of poor health, wellbeing and inclusion outcomes [21,22]. For children, however, work may be a form of child labour or indicative of other adversity, for example experiences of exploitation and abuse, or low socio-economic status which may force the child to contribute to family income. It may also mean that the child is out of education, and girls are more likely to be unfairly impacted. For children, work may therefore mean exposure to hazardous labour, or reduced access to schooling which could exacerbate the negative effects of violence [23–25]. Taken as a whole, this evidence points to the importance of further understanding work as a contextual factor in the lives of children and young people. Yet, we are lacking evidence for whether and how work modifies the association between childhood violence and mental health, and whether there are variations by age and sex.

Previous research has used data from the Violence Against Children Surveys (VACS) to explore the relationship between violence and work [26], and between violence and mental health [27–31]. Yet, no research has investigated the influence of being in work on the relationship between violence and mental health. In this study, we use data from the VACS conducted in Nigeria, Uganda and Colombia among 13–24-year-olds to investigate the association between lifetime experience of physical, sexual or emotional violence, and mental distress in the past 30 days, and assess whether past year paid/unpaid work modifies this association.

## METHODS

### Data sources

The VACS are nationally representative cross-sectional surveys conducted in over 25 low- and middle-income countries. They collect data on experiences of physical, sexual and emotional violence among males and females aged 13–24 years old, in addition to data on risk and protective factors, and consequences of violence. 

Here we used data from the VACS conducted in Nigeria (2014), Uganda (2015), and Colombia (2018). The surveys used a multi-stage cluster sampling design to select only one individual per household. Through a split sample approach, they sampled males and females from different enumeration areas to protect confidentiality and reduce the likelihood of interviewing a survivor and perpetrator within the same area. For the data collection, trained interviewers interviewed young people in a safe and private location, and separately surveyed the head of household to collect household-level socio-economic data. The surveys adhered to World Health Organization (WHO) recommendations on ethics around conducting research on violence against women and with children [32]. Informed consent or assent was obtained for all participants prior to enrolment and interviewers were trained to provide referrals to support services, depending on each respondent’s specific experiences.

### Measures

#### Outcome

The outcome of interest was mental distress, measured using the six-item Kessler Psychological Distress Scale (K6) [33]. Participants were asked six questions on how frequently in the past 30 days they felt nervous; hopeless; restless; so sad that nothing could cheer them up; that everything was an effort; or worthless. There were four response options ranging from ‘none’ to ‘all of the time’, scored between 0 and 4, respectively. Scores for each question were summed and classified using the total score as no mental distress (score of 0–4), moderate mental distress (score of 5–12) or severe mental distress (score of 13–24). Due to small sample sizes and in line with previous literature [34,35], we further classified this measure into a binary variable to represent no mental distress (score of 0–4) or moderate/severe mental distress (score of 5–24). We excluded individuals if they were missing data for any of the six questions from the analysis.

#### Exposure

The exposure of interest was lifetime experience of any physical, sexual, or emotional violence from peers, partners, adult caregivers or other adult relatives, or adults in the community or neighbourhood. In the Nigerian and Ugandan surveys, participants were asked about physical violence for each perpetrator, and the following acts were included in the measure: punching, kicking, whipping or beating with an object; choking, suffocating, attempting to drown, intentionally burning; using or threatening with a knife, gun or other weapon. The Colombia survey contained an additional item to measure physical violence, defined as a perpetrator ever slapping, pushing, shoving, shaking, or intentionally throwing something at the respondent to hurt them.

All surveys defined sexual violence as whether any perpetrator has ever: touched the participant in a sexual way without their permission, but did not try to force them to have sex; tried to make them have sex against their will, but did not succeed; physically forced them to have sex and did succeed; or pressured them to have sex and did succeed.

Emotional violence was defined in the Nigerian and Ugandan surveys as experience of any of three items by a caregiver/adult relative who: told the participant that they were not loved or did not deserve to be loved; said they wished they had never been born or were dead; and ridiculed them or put them down by, for example, saying that they were stupid or useless. The Colombian survey had an additional item: threatened to get rid of the participant.

We constructed a binary variable for experience of any violence, defined by experience of any of physical, sexual or emotional violence by any perpetrator. The numerator included participants who reported violence, and the denominator included participants who reported no experience of physical, sexual or emotional violence, or that they did not know.

#### Covariates

We selected the covariates based on their theoretical relevance to both lifetime violence and mental distress, and we used the socioecological model to guide our selection of individual, relationship, community, and societal factors that may be of theoretical relevance. We included age and household size as continuous variables. Household-level wealth was included as a covariate. We created a wealth index using principal components analysis to combine variables related to household assets, access to water and sanitation facilities, and housing materials, after which we divided the population into wealth quintiles [36]. All other covariates were binary and included: completion of primary school (reference: did not complete primary school), female-headed household (reference: not a female-headed household), parental death (reference: both parents alive), ever married (reference: never married), any work in the past year (reference: no work in the past year). We excluded substance use (alcohol, tobacco smoking, or other drugs) in the past 30 days as a covariate given the possibility that this was a mediator in the relationship between lifetime violence and later mental distress.

We explored whether being in work in the past year was a potential effect modifier of the association between experience of violence and mental distress. In all three surveys, being in work in the past year was defined as any paid or unpaid work in the past 12 months as an employee or self-employed. We constructed a binary variable for work in the past year, with the denominator being all participants of the survey.

### Analysis

We first estimated the prevalence of mental distress, past year paid/unpaid work, lifetime experience of physical, sexual and emotional violence, and any lifetime violence, as well as the proportions of individuals across covariates among the whole population in each country, stratified by sex. We then estimated the prevalence of violence and mental distress stratified by being in paid/unpaid work in the past year, and by sex and age group (13–17-year-olds vs 18–24-year-olds). We used χ^2^ tests and corresponding *P*-values to assess the evidence for a difference in the above-mentioned variables, as well as difference in prevalence of lifetime violence and mental distress by work status.

We then explored the association between lifetime violence and mental distress among 13–24-year-olds. We conducted univariate analyses using separate logistic regression models, with lifetime violence as the exposure and mental distress as the outcome, to obtain crude estimates of the association between lifetime violence and mental distress among 13–24-year-olds in each country. We also performed multivariable analyses using logistic regression, where model building was guided by the socioecological model. We included past-year paid/unpaid work as a covariate in all adjusted models.

Finally, we explored whether being in work in the past year influenced the association between lifetime violence and mental distress. Using the previously-described logistic regression model, we fitted an interaction between work and lifetime violence and used Wald tests to assess the strength of evidence for effect modification.

All analyses were conducted in Stata, version 18 (StataCorp LLC, College Station, Texas, USA) and were survey-weighted to account for the survey design. All analyses were country-specific and stratified by sex given the differing patterns of violence and their mechanisms in males and females, as well as by age (13–17 years vs 18–24 years) given the differences in the roles of work and mechanisms at play for children compared to young adults. We reported adjusted odds ratios (aORs) and 95% confidence intervals (CIs) for all regression models. Given the possibility of certain findings being due to chance, we interpreted both the statistical significance and the pattern of the findings. In presenting our results, we refer to individuals as young men or young women for individuals of any age included in the VACS, and we refer to boys or girls when specifically talking about children (individuals under 18 years of age).

## RESULTS

We included data from 12 712 young people aged 13–24 years from three countries that conducted VACS surveys between 2014–18 [37–39]. Country-specific sample sizes were 4203 individuals in Nigeria (2014), 5804 in Uganda (2015), and 2705 in Colombia (2018).

### Prevalence of violence, mental distress and work

**Figure 1:**
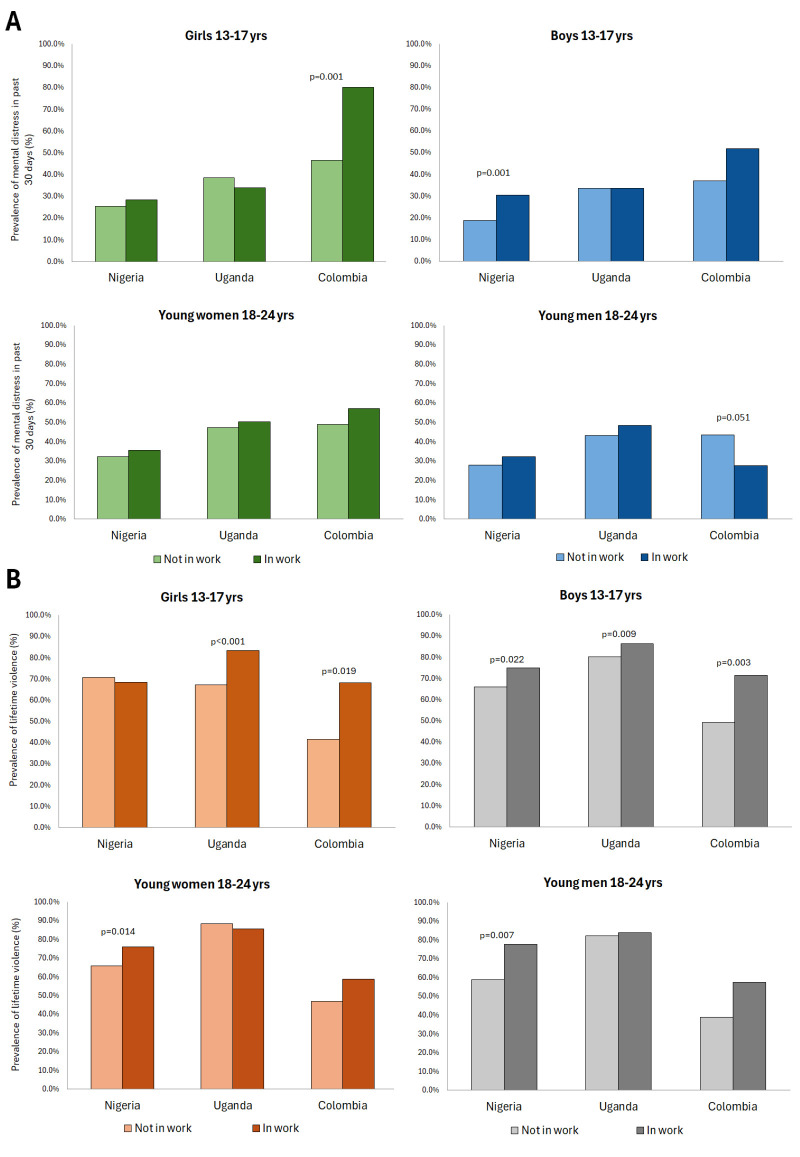
**Panel A.** Prevalence of mental distress by work status in past year. **Panel B.** Prevalence of lifetime violence by work status in past year.

We examined the prevalence of demographics, exposures, modifiers and outcomes by country stratified by sex (**Table 1**) and the prevalence of exposures and outcomes stratified by country, sex, age, and work status (**Figure 1**; Table S1 in the **Online Supplementary Document**). The percentage of young people in paid/unpaid work in the past 12 months was highest in Nigeria (63.7%), followed by Uganda (54.5%) and Colombia (46.9%). In Nigeria and Colombia, being in work in the past year was more common among young men (75.9% and 56.4%, respectively) compared to young women (51.9 and 37.0%, respectively) (*P* < 0.001), while there was little difference in Uganda (young men: 54.6%, young women: 54.2%).

**Table 1:** Prevalence of demographics, exposures, modifiers, and outcomes by country, stratified by sex*

	Nigeria, n (%)				Uganda, n (%)				Colombia, n (%)			
	**Total**	**Female**	**Male**	***P*-value**	**Total**	**Female**	**Male**	***P*-value**	**Total**	**Female**	**Male**	***P*-value**
**Total**	4203 (100)	1766 (50.8)	2437 (49.2)		5804 (100)	3159 (39.28)	2645 (60.7)		2705 (100)	1406 (48.9)	1299 (51.1)	
**Age**				0.032				<0.001				0.900
13–17 y	1847 (43.1)	797 (40.8)	1050 (45.4)		2746 (47.8)	1364 (41.8)	1382 (51.6)		1290 (41.5)	665 (41.7)	625 (41.3)	
18–24 y	2356 (56.9)	969 (59.2)	1387 (54.6)		3058 (52.2)	1795 (58.2)	1263 (48.4)		1415 (58.5)	741 (58.3)	674 (58.7)	
**Completed primary school**	3124 (73)	1213 (68.5)	1911 (77.6)	0.025	3550 (63.2)	1999 (66.8)	1551 (60.8)	0.028	2571 (96.3)	1331 (95.8)	1240 (96.8)	0.449
**Parental death (reference: both parents alive)†**	117 (3.3)	76 (5.2)	41 (1.4)	<0.001	119 (1.9)	89 (2.9)	30 (1.2)	0.001	21 (1.1)	12 (1.7)	9 (0.6)	0.207
**Ever married**	862 (21.3)	558 (31.8)	304 (10.5)	<0.001	1670 (24.4)	1259 (38.2)	411 (15.5)	<0.001	75 (2.5)	56 (3.7)	19 (1.4)	0.098
**Female-headed household†**	750 (18.7)	323 (24)	316 (13.2)	<0.001	1573 (28.8)	837 (30.0)	736 (28.1)	0.394	1232 (56.4)	753 (63.4)	479 (49.5)	0.005
**Number of household members, x̄ (SE)**	6.589 (0.13)	6.39 (0.21)	6.78 (0.20)	0.042	5.83 (0.07)	5.61 (0.13)	5.97 (0.79)	<0.001				
**Paid or unpaid work in past year**	2730 (63.7)	907 (51.9)	1823 (75.9)	<0.001	3210 (54.5)	1728 (54.2)	1482 (54.6)	0.872	1146 (46.9)	499 (37.0)	647 (56.4)	<0.001
**Moderate or severe mental distress†**	1248 (30.4)	537 (31.2)	711 (29.4)	0.454	2382 (41.1)	1022 (39.5)	1360 (43.6)	0.099	1186 (44.0)	724 (52.2)	462 (36.2)	<0.001
**Lifetime violence**												
Any violence	2960 (70.4)	1258 (70.8)	1702 (70.1)	0.819	4839 (83.2)	2642 (82.5)	2197 (83.6)	0.542	1350 (52.0)	669 (49.7)	681 (54.4)	0.369
Physical violence	2537 (59.6)	1058 (58.4)	1479 (60.8)	0.421	4331 (74.0)	2286 (68.0)	2045 (78.0)	<0.001	1106 (41.4)	504 (35.9)	602 (46.7)	0.047
Sexual violence	1093 (27.3)	609 (35.2)	484 (19.2)	<0.001	1998 (31.4)	1381 (43.9)	617 (23.3)	<0.001	441 (16.8)	285 (21.2)	156 (12.7)	0.034
Emotional violence	1073 (24.9)	388 (21.7)	685 (28.1)	0.008	2365 (40.6)	1307 (39.1)	1058 (41.6)	0.235	513 (19.3)	307 (22.8)	206 (15.9)	0.053

*Percentages are survey weighted. Sample sizes represent the unweighted sample.

†Missing data for variables: parental death (n = 163 for Nigeria, n = 325 for Uganda), female-headed household (n = 106 for Nigeria, n = 8 for Uganda, n = 403 for Colombia), and mental distress (n = 259 for Nigeria, n = 146 for Uganda, and n = 30 for Colombia).

#### Prevalence of mental distress

The prevalence of past 30-day mental distress was highest in Colombia (44.0%), followed by Uganda (41.1%) then Nigeria (30.4%) (**Figure 1**, Panel A). Mental distress was more prevalent among young women (52.2%) than men (36.2%) in Colombia (*P* < 0.001), but there was no evidence of a difference between young men and women in Nigeria (31.2% vs 29.4%, *P* = 0.454) and Uganda (39.5% vs 43.6%, *P* = 0.099).

Among young women, there was strong evidence of a higher prevalence of mental distress among 13–17-year-old girls in Colombia who had been in work in the past year (80.2%) compared to those not in work in the past year (46.6%) (*P* < 0.001). There were few other differences in the prevalence of mental distress by work status among young women, though it tended to be slightly higher among 18–24-year-olds in work in the past year in all three countries.

Among young men, there was evidence of a higher prevalence of mental distress among 13–17-year-old boys in work the past year in Nigeria (30.6%) compared to those not in work in the past year (18.8%) (*P* = 0.001). There was also some evidence of a higher prevalence of mental distress among 18–24-year-old young men in Colombia who were not in work in the past year (43.6%) compared to those in work in the past year (27.6%) (*P* = 0.051). There were few differences in the prevalence of mental distress by work status in other countries and population groups. Data on mental distress was missing for 259, 146, and 30 individuals in Nigeria, Uganda and Colombia data, respectively.

#### Prevalence of violence

The prevalence of lifetime violence among young people aged 13–24 years was highest in Uganda (83.2%), followed by Nigeria (70.4%) and Colombia (52.0%) (**Figure 1**, Panel B). Across all three countries, physical violence was the most common form of violence, the prevalence of physical violence was higher among young men than women, and sexual violence was higher among young women.

The prevalence of ever experiencing physical, sexual, or emotional violence by work status in the past year varied by sex, age group and country. Among 13–17-year-old girls, there was a higher prevalence of lifetime violence among those in work in the past year compared to those not in work in the past year in Uganda (67.2% vs 83.3%, *P* < 0.001) and Colombia (41.5% vs 68.1%, *P* = 0.019). This was driven mainly by a higher prevalence of physical violence in Uganda and that of sexual violence in Colombia. Among 18–24-year-old young women, the prevalence of violence was higher among those in work in the past year compared to those not in work in the past year in Nigeria (65.8% vs 76.0%, *P* = 0.014), driven mainly by experience of physical and sexual violence.

Among 13–17 boys in all three countries, the prevalence of any lifetime violence was higher among those in work in the past year compared to those not in work in the past year (Nigeria: 65.9% vs 74.8%, *P* = 0.022; Uganda: 80.3% vs 86.5%, *P* = 0.009; Colombia: 49.3% vs 71.6%, *P* = 0.003). This was mainly driven by physical violence in Uganda and Colombia, and both physical and emotional violence in Nigeria. We saw similar results among young men aged 18–24 years with a higher prevalence of lifetime violence among those in work in the past year in Nigeria (59.0% vs 70.6%, *P* = 0.007) and Colombia (38.8% vs 57.4%, *P* = 0.027), driven by an increase of physical violence.

### Work as an effect modifier of the association between lifetime violence and mental distress

**Figure 2:**
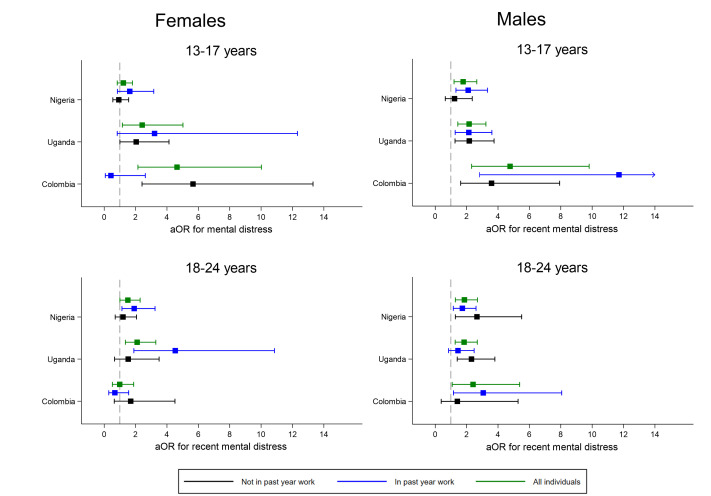
Associations between lifetime violence and past 30-day mental distress among those not in work and those in work.

We then explored the association between lifetime experience of any violence and past 30-day mental distress (Tables S2 and S3 in the **Online Supplementary Document**) and if and how being in work in the past year modified this association (**Figure 2**; Table S4 in the **Online Supplementary Document**).

Among 13–17-year-old girls, there was very strong evidence in Colombia for an association between lifetime violence and recent mental distress (aOR = 4.95; 95% CI = 2.29–10.68, *P* < 0.001). There was also strong evidence that work in the past year modified this relationship (*P*-value for interaction = 0.014), with a strong association among those not in work in the past year (aOR = 6.12; 95% CI = 2.60–14.41, *P* < 0.001), but no association between violence and distress among those in work in the past year (aOR = 0.42; 95% CI = 0.07–2.57, *P* = 0.349). In Uganda, there was evidence for an association between experiencing lifetime violence and recent mental distress (aOR = 2.72; 95% CI = 1.26–5.88, *P* = 0.011) and work in the past year did not modify this relationship (interaction *P* = 0.661). There was no evidence for an association between violence and mental distress among girls in Nigeria (aOR = 1.30; 95% CI = 0.84–2.00, *P* = 0.243).

Among 18–24-year-old women, there was strong evidence for an association between lifetime violence and recent mental distress in Uganda (aOR = 2.56; 95% CI = 1.33–4.94, *P* = 0.005). There was also some evidence that work in the past year modified this association (*P*-value for interaction = 0.058), where the odds of mental distress was higher among those in work in the past year (aOR = 4.23; 95% CI = 1.78–10.03, *P* = 0.001) compared to those not in work in the past year (aOR = 1.46; 95% CI = 0.64–3.37, *P* = 0.368). There was also strong evidence for an association between violence and mental distress among 18–24-year-olds in Nigeria (aOR = 1.87; 95% CI = 1.20–2.93, *P* = 0.006). While we found no evidence for work being an effect modifier in Nigeria, there was strong evidence for an association between lifetime violence and recent mental distress among those in work in the past year (aOR = 2.06; 95% CI = 1.19–3.56, *P* = 0.010), but not among those not in work in the past year (aOR = 1.70; 95% CI = 0.94–3.05, *P* = 0.078). We found no evidence for an association between violence and mental distress among 18–24-year-olds in Colombia (aOR = 1.00; 95% CI = 0.52–1.89, *P* = 0.989).

Among 13–17-year-old boys, there was strong evidence for an association between lifetime violence and recent mental distress in all three countries. In Nigeria, the odds of recent mental distress were 1.93 times among boys who had ever experienced violence (95%CI 1.17–3.19, *P* = 0.011) compared to boys not experiencing violence. There was also evidence that work modified this association (*P*-value for interaction = 0.045), with a higher odds of mental distress among those in work in the past year (aOR = 2.10; 95% CI = 1.32–3.33, *P* = 0.002) compared to those not in work in the past year (aOR = 0.99; 95% CI = 0.46–2.13, *P* = 0.979). In Uganda and Colombia, there was strong evidence among boys that experiencing lifetime violence increased the odds of experiencing recent mental distress (Uganda: aOR = 2.29; 95% CI = 1.49–3.52, *P* < 0.001; Colombia: aOR = 4.85; 95% CI = 2.35–10.01, *P* < 0.001). There was no evidence that being in work in the past year modified this association in Uganda or Colombia (Uganda: *P*-value for interaction = 0.861; Colombia: *P*-value for interaction = 0.123). However, in Colombia, the OR tended to be higher among those in work in the past year (aOR = 12.40; 95% CI = 2.89–53.28, *P* = 0.001), compared to those not in work in the past year (aOR = 3.55; 95% CI = 1.60–7.88, *P* = 0.002).

Among 18–24-year-old young men, there was similarly strong evidence of an association between lifetime experience of violence and recent mental distress in Nigeria (aOR = 1.90; 95% CI = 1.25–2.90, *P* = 0.003), Uganda (aOR = 1.91; 95% CI = 1.30–2.82, *P* = 0.001), and Colombia (aOR = 2.11; 95% CI = 1.01–4.42, *P* = 0.047). There was no evidence that being in work in the past year modified this association in any country (Nigeria: *P*-value for interaction = 0.088; Uganda: *P*-value for interaction = 0.157; Colombia: *P*-value for interaction = 0.357). While we found no evidence for work being an effect modifier in Uganda, associations did differ by work status, with very strong evidence of an association among those not in work in the past year (aOR = 2.48; 95% CI = 1.48–4.15, *P* = 0.001), but not among those in work in the past year (aOR = 1.46; 95% CI = 0.85–2.51, *P* = 0.171). We saw an opposite pattern in Colombia, where there was evidence for an association among those in work in the past year (aOR = 2.66; 95% CI = 1.14–6.18, *P* = 0.023), but not among those not in work in the past year (aOR = 1.30; 95% CI = 0.35–4.87, *P* = 0.699).

We could not examine results stratified by paid and unpaid work, as this was not measured in the Nigerian and Ugandan surveys. However, we found that being in paid compared to unpaid work in Colombia did not modify the association between lifetime violence and work in the past year (Table S5 in the **Online Supplementary Document**).

## DISCUSSION

This multi-country study draws on VACS data from over 12 000 individuals aged 13–24 years old in Nigeria, Uganda, and Colombia. We found a high prevalence of physical, sexual, and emotional violence across the lifetime among children and young people, often higher among those in past year work, and a consistently strong association between violence and mental distress among young men in all countries and of all ages, and for girls in Uganda and Colombia and 18–24-year-old young women in Nigeria and Uganda. This is in line with the large body of evidence documenting the strong association between violence and later mental health problems [7,8].

In line with previous literature [26], the prevalence of lifetime violence among girls and boys in all three countries, except for girls in Nigeria, was higher among those in work in the past year than those not in work in the past year. Among young people, there was also a higher prevalence of lifetime violence among young men aged 18–24 years in Colombia and both 18–24-year-old men and women in Nigeria who were in work in the past year. We found few differences in the prevalence of recent mental distress by past year work status.

Our findings highlight differences by country, sex, and age group in terms of whether being in work in the past year modified the association between lifetime violence and mental distress. We found evidence in Colombia that being in work in the past year reduced the risk of mental distress among 13–17-year-old girls who had experienced violence compared to girls who had not experienced violence. In Nigeria, meanwhile, being in work increased the risk of mental distress among 13–17-year-old boys who had experienced violence. While we did not find evidence of effect modification by in other countries and age groups, there were patterns towards past year work exacerbating the risk of mental distress compared to those not in work in the past year among young women of all ages in Nigeria and Uganda who had ever experienced violence. Our findings suggest that work may modify the relationship between violence and mental health among young women. In contrast, among young men, there were trends towards work being protective among 18–24-year-old young men in Nigeria and Uganda, and exacerbating the risk of distress among men in Colombia. Our findings therefore suggest that work may modify the relationship between violence and mental health among young women and men.

In Nigeria and Uganda, work appeared to have the opposite influence on the violence-mental distress association for 18–24-year-old men and women. Though not an effect modifier, being in work in the past year seemed to exacerbate the risk of mental distress among young women who had experienced violence, but was slightly protective among young men in Nigeria and Uganda. Job formality and type of sector may partly explain these differences by sex, given that young men and women are often concentrated in different job sectors. Evidence suggests that, in sub-Saharan Africa, young women are more likely than young men to be self-employed or domestic workers, and less likely to be employers or employees, which is likely to result in lower pay and less security [40,41]. These informal sectors are also less likely to provide protection and support to vulnerable people. Evidence suggests lower income and less job security is associated with worse mental health, particularly among young people [18,42,43], all of which may contribute to the gender differences observed in our results. Young women and men also experience different forms of violence in the workplace [44], which may influence the risk of mental distress [27]. The work environment, surrounding norms, and experiences of being in work are also gendered and may explain differing mental health outcomes among men and women [45].

The findings in Colombia showed patterns distinct from those in Nigeria and Uganda and were generally in the opposite direction. Among young women in Colombia who had experienced violence, being in work in the past year seemed to protect against developing mental distress, while among young men, it tended to exacerbate the risk of mental distress. The findings among women are in line with previous evidence around the protective nature of work, including protecting against the development of depression and improving general mental health [17], and earning a higher income is associated with improved mental health, particularly in low- and middle-income settings [18]. The differing findings between women and men suggest that workplace exposures may either vary by sex or may have different impacts on young men and women. Type of work and differing sectors between countries may also be important in explaining these differences, where work may act as a protective context among young women but exacerbate poor mental health among young men. Each country also has a different policy context and legislation for children’s work, including differences in the minimum age for full time work, which may be important in explaining differences in our findings between countries.

Our findings show trends towards work exacerbating the effects of lifetime violence for children aged 13–17 years. It may be that children who are in work are at an economic disadvantage and working out of necessity, and it may mean they are out of education and training. Work which is classified as child labour is also more likely to be under regulated with more limited protection for children. These factors may also exacerbate the effects of experiencing violence. Type of work is also likely to be important. For example, there is a high prevalence of children working in hazardous worksites [26], more likely among boys than girls, which may exacerbate the effects of violence for children. There is also evidence from child labour surveys across multiple countries that children working in hazardous sectors have poorer health than other working children, a higher prevalence of mental and behavioural disorders, and that they experience higher levels of abuse [46,47]. The trends across our results suggest work may also have slightly less of an effect among boys in all countries compared to girls, which may be explained by the more harmful nature of the workplace, pay, or sectors for girls compared to boys, thereby further exacerbating the risk of mental distress [44]. In contrast, young people aged 18–24 years may be working under different conditions to children where work may not be replacing education, and where they may have increased ability to negotiate access to work compared to children.

The strengths of this study include the use of national data from three countries, each with a large sample size and comparable data. We also stratified our analysis by age and sex to highlight the distinct relationships seen in different population groups. This study presents some of the first evidence around the importance of the work context and how it mitigates, entrenches, or exacerbates the risk of mental distress among those who experience violence.

However, there are several limitations to this study. The first relates to the measurement of work and violence in the VACS. We were unable to consider the specific characteristics of work, including the type of work, seasonality, job security, and whether it is unpaid or paid, all of which may influence whether work is protective or harmful. While the Colombia VACS does measure paid vs unpaid work, sample sizes for the interaction analyses were small when age- and sex-stratified, and results would have been incomparable to the Nigerian and Ugandan VACS, where paid vs unpaid work is not measured. We did, however, conduct an additional analysis with the Colombian VACS data to find few differences in the association between lifetime violence and mental distress among those in paid compared to unpaid work. While it would have also been useful to look at hazardous work separately as an effect modifier, this was not possible, as the data was not comparable across countries and the sample sizes of those in hazardous work were too small. We were therefore unable to provide more nuanced information on whether specific types of work may mitigate or exacerbate the risk of mental distress compared to other types. In relation to the measurement of violence, the Colombian VACS included an additional item for some types of violence, compared to Nigeria and Uganda. However, as the surveys shared most items, it is unlikely that this significantly affected the results. There was also no measure of lifetime workplace violence in the VACS, which would have been useful in furthering our understanding of the pathways through which work may influence the relationship between violence and mental distress.

A second limitation is the use of cross-sectional data. We looked at experience of lifetime violence and the experience of mental distress in the past 30 days to maximise the likelihood that the violence occurred prior to mental distress. While we cannot rule out the possibility of a bidirectional relationship, where the violence occurred recently following mental distress, this is unlikely for the majority of participants given the timeframe of exposure and outcome.

A third limitation relates to our strategy for analysis and modelling. We classified violence into a binary measure and therefore did not account for the severity, frequency, perpetrator of violence, and context in which it occurs (e.g. the workplace, school, home or neighbourhood). Context and perpetrator are likely to be particularly important in this study, given that we explored whether the work context is protective or harmful. We also did not account for what young people are doing who are not in work – for example, whether they are in school or out of education and training. These population groups are likely to vary in characteristics that may influence risk of mental distress. While we adjusted for variables we believed to be theoretically relevant in our models, there may also be unmeasured and residual confounding. The sample sizes were also small for some of the effect modification analyses, as the numbers of individuals who experienced mental distress and lifetime violence and were in work in the past year was low for some subgroups. We therefore had wide confidence intervals for some results.

A fourth limitation relates to the possibility that being in work in the past year could be a mediator of the relationship between lifetime violence and recent mental distress. While evidence does suggest violence may influence later work outcomes [6,48,49] and we cannot rule out this possibility, other studies found that work can increase the risk of experiencing violence in childhood [24,25,44]. These links are complex and there is currently no conclusive evidence on the direction of the relationships.

Our findings have important implications for research, as they highlight the importance of VACS data for exploring the experience of violence and adverse outcomes, and how different contexts may influence this relationship. There are few data sources that collect data on violence, mental health, and work at a national scale across countries and among young people specifically.

Our findings also highlight the importance of work as a context that young people spend time in and are exposed to. Most research focuses on the home and school environment, with a lack of evidence around the work context. Our findings suggest that being in work can both mitigate and exacerbate the risk of mental distress following experience of violence depending on age, sex and country. Further research should aim to understand why this is. For example, reasons for work being protective may be related to earning an income, being connected with others, receiving support from the workplace, or being exposed to reduced violence due to being out of a harmful home or school context. Simultaneously, work may be harmful if individuals are exposed to violence in the workplace or experience poor working conditions that further exacerbate the risk of mental distress. It is therefore important to look at the perpetrators of violence and contexts in which violence is occurring, including measurement of workplace violence specifically, as this will affect the protective or harmful nature of the workplace.

Research should also make use of longitudinal data to provide more accurate information on the association between experiencing violence and later experiencing mental distress, with attention to the role of contextual factors in mitigating, interrupting or exacerbating these associations. Such efforts could offer opportunities to shape contexts to both prevent violence and prevent the short- and long-term impacts of violence as children grow up.

There are also important implications for data collection in the VACS, which should collect more detailed information on the specific nature of work that is comparable across countries. This could include, for example, whether said work is unpaid or paid, whether it is in a specific sector, the number or hours worked, and other factors that may affect how protective or harmful work can be as a context for young people.

Although further research is needed, our findings show that the contexts of work matter for young people. Governments and both public and private sectors need to ensure that workplaces are safe and secure for young people, and do not further exacerbate the risk of mental distress. Given that we know that those who experience violence in childhood are much more likely to develop mental distress, it is also important to ensure that they know how and can support the mental health of this population. Efforts should also be made to develop and integrate interventions into workplaces to support those who have experienced violence. The clear differences by age, sex, and country highlight the importance of having context-specific and targeted interventions that are gender responsive and tailored to young people of various ages and life stages.

## CONCLUSIONS

Taken together, our findings suggest that being in work can mitigate or exacerbate the risk of mental distress among young people who have experienced violence depending on the ages, life stages and sex of young people. While further research is required to better understand the role of workplaces and workplace violence in young people’s lives in being either protective or harmful, this study highlights the important influence that the work context can have on young people.

## Additional material


Online Supplementary Document
