# Supplementary material for: Does work modify the relationship between violence and mental health among young people? Evidence from the Violence Against Children Surveys in Uganda, Nigeria and Colombia
Source: J Glob Health. 2024 Nov 22;14:04232. doi: 10.7189/jogh.14.04232 (PMC11583282; doi:10.7189/jogh.14.04232)
Supplement: Online Supplementary Document [file jogh-14-04232-s001.pdf]

Supplementary Files

Table S1. Prevalence of lifetime violence and mental distress stratified by sex, age and past year work status

Table S1A: Among young women

	Girls 13-17 yrs									Young women 18-24 yrs								
	Not in work (past year; paid or unpaid)				In work (past year; paid or unpaid)					Not in work (past year; paid or unpaid)				In work (past year; paid or unpaid)				
	n	%	95% CI		n	%	95% CI		p-value	n	%	95% CI		n	%	95% CI		p-value
**Nigeria**																		
Lifetime violence																		
Any violence	306	70.7%	64.3%	76.4%	244	68.6%	62.0%	74.5%	0.589	287	65.8%	58.7%	72.3%	421	76.0%	69.6%	81.4%	0.014
Physical violence	269	62.6%	56.0%	68.8%	204	56.7%	49.0%	64.1%	0.191	233	52.1%	45.1%	59.0%	352	61.5%	54.2%	68.2%	0.032
Sexual violence	112	25.9%	20.9%	31.6%	90	27.6%	21.0%	35.3%	0.708	159	34.7%	29.3%	40.6%	248	46.1%	40.1%	52.3%	0.002
Emotional violence	79	17.0%	13.2%	21.6%	81	23.4%	17.6%	30.4%	0.082	87	19.8%	15.3%	25.3%	141	25.2%	19.7%	31.7%	0.136
Moderate/severe mental distress	96	25.4%	19.1%	32.9%	99	28.5%	22.7%	35.2%	0.497	137	32.4%	26.8%	38.5%	205	35.6%	29.9%	41.7%	0.448
**Uganda**																		
Lifetime violence																		
Any violence	463	67.2%	58.1%	75.2%	642	83.3%	77.0%	88.2%	<0.001	671	88.3%	83.9%	91.6%	866	85.7%	80.1%	89.9%	0.401
Physical violence	406	59.8%	51.3%	67.7%	573	72.1%	64.6%	78.6%	0.014	558	70.3%	62.7%	77.0%	749	67.6%	60.5%	74.0%	0.594
Sexual violence	208	33.0%	24.3%	43.1%	269	32.5%	26.7%	39.0%	0.931	407	57.4%	50.6%	64.0%	497	47.0%	41.4%	52.6%	0.030
Emotional violence	235	33.3%	26.2%	41.3%	327	34.9%	29.4%	40.9%	0.722	302	43.3%	36.5%	50.3%	443	42.0%	36.5%	47.8%	0.795
Moderate/severe mental distress	206	38.6%	29.1%	49.1%	204	34.1%	27.8%	40.9%	0.406	386	47.3%	40.2%	54.5%	524	50.4%	44.2%	56.6%	0.525
**Colombia**																		
Lifetime violence																		
Any violence	204	41.5%	30.9%	53.1%	59	68.1%	47.4%	83.5%	0.019	154	47.1%	32.63	62.0%	252	58.7%	49.0%	67.8%	0.199
Physical violence	134	30.3%	19.9%	43.3%	49	46.3%	27.6%	66.1%	0.163	121	30.8%	21.23	42.4%	200	45.0%	36.0%	54.3%	0.047
Sexual violence	73	13.8%	8.8%	21.1%	28	31.1%	16.4%	50.8%	0.036	62	28.6%	14.7	48.2%	122	22.2%	15.2%	31.3%	0.402
Emotional violence	109	19.4%	11.8%	30.1%	30	34.8%	18.6%	55.5%	0.124	67	18.6%	10.1	31.7%	101	28.3%	19.3%	39.4%	0.150
Moderate/severe mental distress	286	46.6%	36.5%	57.0%	59	80.2%	63.8%	90.3%	0.001	160	49.0%	38.1%	60.0%	219	57.1%	47.1%	66.5%	0.285

Percentages and 95% CIs are survey weighted. Sample sizes represent the unweighted sample.

Table S1B: Among young men

	Boys 13-17 yrs									Young men 18-24 yrs								
	Not in work (past year; paid or unpaid)				In work (past year; paid or unpaid)					Not in work (past year; paid or unpaid)				In work (past year; paid or unpaid)				
	n	%	95% CI		n	%	95% CI		p-value	n	%	95% CI		n	%	95% CI		p-value
**Nigeria**																		
Lifetime violence																		
Any violence	241	65.9%	59.3%	72.0%	521	74.8%	69.8%	79.2%	0.022	149	59.0%	50.2%	67.3%	791	70.6%	66.5%	74.4%	0.007
Physical violence	210	57.1%	50.4%	63.6%	465	65.2%	59.6%	70.3%	0.054	117	45.4%	37.8%	53.4%	687	62.3%	57.9%	66.5%	<0.001
Sexual violence	48	12.8%	9.2%	17.6%	97	13.9%	11.0%	17.6%	0.675	58	24.4%	17.9%	32.3%	281	23.7%	20.0%	27.8%	0.855
Emotional violence	89	24.1%	19.0%	30.1%	226	32.2%	27.3%	37.6%	0.030	54	22.6%	17.3%	29.1%	316	27.8%	23.9%	32.1%	0.188
Moderate/severe mental distress	60	18.8%	14.2%	24.6%	202	30.6%	26.1%	35.5%	0.001	71	27.9%	22.0%	34.7%	378	32.3%	28.2%	36.8%	0.230
**Uganda**																		
Lifetime violence																		
Any violence	402	80.3%	75.9%	84.0%	121	86.5%	83.4%	89.1%	0.009	532	82.4%	78.8%	85.5%	515	83.9%	80.0%	87.1%	0.545
Physical violence	380	76.1%	71.4%	80.2%	710	82.3%	78.9%	85.2%	0.016	474	74.2%	70.3%	77.7%	481	77.8%	73.0%	81.9%	0.218
Sexual violence	89	17.0%	13.8%	20.8%	152	18.1%	15.0%	21.7%	0.663	192	29.5%	25.6%	33.7%	184	29.2%	25.1%	33.5%	0.914
Emotional violence	185	40.5%	35.2%	46.0%	335	39.5%	36.0%	43.0%	0.747	288	45.9%	40.8%	51.0%	250	40.9%	36.4%	45.5%	0.144
Moderate/severe mental distress	168	33.7%	28.7%	39.1%	282	33.6%	29.8%	37.5%	0.962	280	43.2%	38.8%	47.6%	292	48.5%	43.2%	53.8%	0.120
**Colombia**																		
Lifetime violence																		
Any violence	218	49.3%	41.4	57.2%	92	71.6%	57.8%	82.6%	0.003	74	38.8%	25.62	53.8%	297	57.4%	47.7%	66.7%	0.027
Physical violence	180	41.9%	34.3	49.9%	84	56.6%	34.2%	76.6%	0.212	67	35.0%	22.29	50.3%	271	51.0%	38.9%	63.0%	0.075
Sexual violence	33	9.3%	4.7	17.6%	19	7.5%	3.5%	15.2%	0.657	21	12.6%	6.593	22.8%	83	16.7%	10.6%	25.2%	0.403
Emotional violence	71	15.6%	10.0	23.4%	35	38.2%	22.1%	57.2%	0.008	20	6.7%	3.3	13.0%	80	12.4%	7.9%	19.0%	0.131
Moderate/severe mental distress	150	37.0%	26.6%	48.6%	60	51.9%	35.8%	67.6%	0.129	67	43.6%	28.9%	59.4%	185	27.6%	20.4%	36.1%	0.051

Percentages and 95% CIs are survey weighted. Sample sizes represent the unweighted sample.

Table S2. Crude associations between lifetime violence and past 30 day mental distress

	Females					Males					All individuals				
	Total n in model	aOR	95% CI		p-value	Total n in model	aOR	95% CI		p-value	Total n in model	aOR	95% CI		p-value
**All individuals**															
Nigeria	1,672	1.55	1.12	2.15	0.009	2,272	1.83	1.37	2.44	<0.001	3,944	1.68	1.35	2.09	<0.001
Uganda	3,094	2.28	1.39	3.74	0.001	2,564	2.03	1.53	2.68	<0.001	5,658	2.12	1.62	2.76	<0.001
Colombia	1,388	2.30	1.42	3.73	0.001	1,287	2.26	1.33	3.84	0.003	2,675	2.17	1.54	3.07	<0.001
**13-17 year olds**															
Nigeria	740	1.15	0.75	1.77	0.527	960	1.94	1.23	3.05	0.004	1,700	1.48	1.09	2.01	0.012
Uganda	1,323	1.92	0.75	4.89	0.172	1,330	2.11	1.42	3.13	<0.001	2,653	1.98	1.21	3.26	0.007
Colombia	657	5.10	2.47	10.51	<0.001	618	4.18	2.03	8.61	<0.001	1,275	4.21	2.58	6.87	<0.001
**18-24 year olds**															
Nigeria	932	1.86	1.24	2.78	0.003	1,312	1.79	1.24	2.58	0.002	2,244	1.83	1.40	2.41	<0.001
Uganda	1,771	2.35	1.26	4.39	0.008	1,234	2.03	1.39	2.96	<0.001	3,005	2.16	1.55	3.02	<0.001
Colombia	731	1.34	0.76	2.38	0.316	669	1.42	0.68	2.95	0.349	1,400	1.45	0.87	2.20	0.170

Percentages and 95% CIs are survey weighted. Sample sizes represent the unweighted sample.

Table S3. Adjusted associations between lifetime violence and past 30 day mental distress

	Females					Males					All individuals				
	Total n in model	aOR	95% CI		p-value	Total n in model	aOR	95% CI		p-value	Total n in model	aOR	95% CI		p-value
**All individuals**															
Nigeria	1,588	1.64	1.17	2.30	0.004	2,036	1.90	1.36	2.66	<0.001	3,624	1.73	1.37	2.18	<0.001
Uganda	2,852	2.31	1.37	3.90	0.002	2,415	1.99	1.48	2.68	<0.001	5,267	2.09	1.58	2.75	<0.001
Colombia	1,156	1.93	1.14	3.25	0.014	1,059	2.95	1.72	5.06	<0.001	2,215	2.25	1.53	3,31	<0.001
**13-17 year olds**															
Nigeria	714	1.30	0.84	2.00	0.243	871	1.93	1.17	3.19	0.011	1,585	1.57	1.13	2.18	0.007
Uganda	1,251	2.72	1.26	5.88	0.011	1,279	2.29	1.49	3.52	<0.001	2,530	2.23	1.37	3.63	0.001
Colombia	539	4.95	2.29	10.68	<0.001	535	4.85	2.35	10.01	<0.001	1,078	4.65	2.69	8.04	<0.001
**18-24 year olds**															
Nigeria	874	1.87	1.20	2.93	0.006	1,165	1.90	1.25	2.90	0.003	2,039	1.85	1.37	2.49	<0.001
Uganda	1,601	2.56	1.33	4.94	0.005	1,136	1.91	1.30	2.81	0.001	2,737	2.08	1.48	2.92	<0.001
Colombia	614	1.00	0.52	1.89	0.989	523	2.11	1.01	4.42	0.047	1,137	1.31	0.80	2.15	0.278

Percentages and 95% CIs are survey weighted. Sample sizes represent the unweighted sample.
Covariates included age, completion of primary school, female-headed household, parental death, ever married, any past year work, wealth quintile. In Nigeria and Uganda, household size was also included as a covariate.

Table S4. Multivariable analysis of lifetime violence and past 30 day mental distress among those not in work and in work

	Females										Males									
	Not in work (past year)					In work (past year)				p-value for interaction	Not in work (past year)					In work (past year)				p-value for interaction
	n	aOR	95% CI		p-value	aOR	95% CI		p-value		n	aOR	95% CI		p-value	aOR	95% CI		p-value	
**All individuals**																				
Nigeria	1,588	1.45	0.92	2.29	0.108	1.84	1.16	2.91	0.01	0.460	2,036	1.80	1.04	3.12	0.037	1.93	1.35	2.77	<0.001	0.803
Uganda	2,852	1.58	0.86	2.91	0.138	3.66	1.70	7.86	0.001	0.051	2,415	2.25	1.50	3.39	<0.001	1.77	1.21	2.59	0.003	0.369
Colombia	1,156	3.72	1.90	7.29	<0.001	0.68	0.32	1.43	0.308	<0.001	1,059	2.22	1.05	4.70	0.038	3.93	1.76	8.79	0.001	0.326
**13-17 year olds**																				
Nigeria	714	1.17	0.65	2.10	0.599	1.43	0.70	2.93	0.328	0.686	871	0.99	0.46	2.13	0.979	2.10	1.32	3.33	0.002	0.045
Uganda	1,251	2.43	1.12	5.29	0.025	3.24	0.88	11.94	0.078	0.661	1,279	2.21	1.26	3.90	0.006	2.36	1.35	4.13	0.003	0.861
Colombia	539	6.12	2.60	14.41	<0.001	0.42	0.07	2.57	0.349	0.014	535	3.55	1.60	7.88	0.002	12.40	2.89	53.28	0.001	0.123
**18-24 year olds**																				
Nigeria	874	1.70	0.94	3.05	0.078	2.06	1.19	3.56	0.010	0.592	1,165	3.56	1.62	7.81	0.002	1.69	1.07	2.66	0.024	0.088
Uganda	1,623	1.46	0.64	3.37	0.368	4.23	1.78	10.03	0.001	0.058	1,136	2.48	1.48	4.15	0.001	1.46	0.85	2.51	0.171	0.157
Colombia	614	1.76	0.67	4.62	0.248	0.67	0.29	1.53	0.339	0.128	523	1.30	0.35	4.87	0.699	2.66	1.14	6.18	0.023	0.357

Percentages and 95% CIs are survey weighted. Sample sizes represent the unweighted sample.
Covariates included age, completion of primary school, female-headed household, parental death, ever married, any past year work, wealth quintile. In Nigeria and Uganda, household size was also included as a covariate.

Table S5. Association between lifetime experience of violence and mental distress among those in unpaid versus paid work in Colombia

	Females											Males										
	Unpaid work (past year)					Paid work (past year)					p-value for interaction	Unpaid work (past year)					Paid work (past year)					p-value for interaction
	n	aOR	95% CI		p-value	n	aOR	95% CI		p-value		n	aOR	95% CI		p-value	n	aOR	95% CI		p-value	
All individuals	441	0.61	0.01	26.55	0.795	441	2.65	0.26	27.07	0.411	0.275	539	30.56	2.14	436.85	0.012	539	12.27	2.01	74.84	0.007	0.242
13-17 year olds	76	0.00	0.00	0.01	<0.001	76	1.00	-	-	-	-	133	3.18	0.04	236.25	0.598	133	5.53	0.43	71.77	0.190	0.506
18-24 year olds	364	2.98	0.1	91.90	0.532	364	3.01	0.31	29.65	0.344	0.219	400	79.73	7.93	801.57	<0.001	400	1	-	-	-	-

Percentages and 95% CIs are survey weighted. Sample sizes represent the unweighted sample.
Covariates included age, completion of primary school, female-headed household, parental death, ever married, any past year work, wealth quintile. In Nigeria and Uganda, household size was also included as a covariate.
